# Graphene-based surface heater for de-icing applications†

**DOI:** 10.1039/c8ra02567c

**Published:** 2018-05-08

**Authors:** Nazmul Karim, Minglonghai Zhang, Shaila Afroj, Vivek Koncherry, Prasad Potluri, Kostya S. Novoselov

**Affiliations:** National Graphene Institute (NGI), The University of Manchester Booth Street East M13 9PL Manchester UK mdnazmul.karim@manchester.ac.uk; Northwest Composites Centre, School of Materials, The University of Manchester James Light Hill Building, 78 Sackville St M1 3BB Manchester UK; School of Physics & Astronomy, The University of Manchester Oxford Road M13 9PL Manchester UK

## Abstract

Graphene-based de-icing composites are of great interest due to incredible thermal, electrical and mechanical properties of graphene. Moreover, current technologies possess a number of challenges such as expensive, high power consumption, limited life time and adding extra weight to the composites. Here, we report a scalable process of making highly conductive graphene-based glass fibre rovings for de-icing applications. We also use a scalable process of making graphene-based conductive ink by microfluidic exfoliation technique. The glass fibre roving is then coated with graphene-based conductive inks using a dip-dry-cure technique which could potentially be scaled up into an industrial manufacturing unit. The graphene-coated glass roving demonstrates lower electrical resistances (∼1.7 Ω cm^−1^) and can heat up rapidly to a required temperature. We integrate these graphene-coated glass rovings into a vacuum-infused epoxy–glass fabric composite and also demonstrate the potential use of as prepared graphene-based composites for de-icing applications.

## Abstract 2

We report a scalable process of making highly conductive graphene-based glass fibre rovings and their integration into a vacuum infused epoxy–glass fabric composite. We then demonstrate the potential use of as prepared composites for de-icing applications.

## Graphical abstract

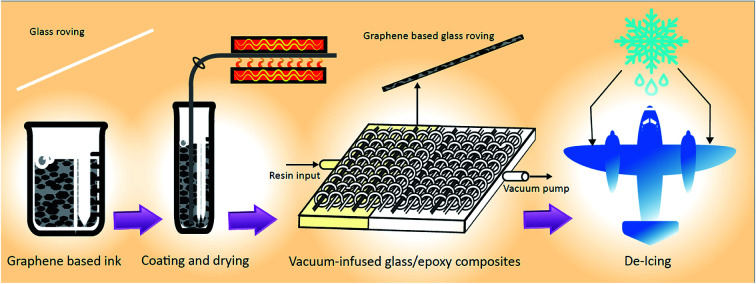

## 1. Introduction

Recent years have seen significant interest in developing smart de-icing systems due to problems associated with the ice formation in many industrial applications such as aircrafts,^1,2^ wind turbines,^3^ power transmission lines^4^ and telecommunications.^5^ Ice accretion can cause huge disruptions to day to day activities of human life^6^ and performance degradation of the structure.^7^ For example, ice accumulation on planes can change the aerodynamic performance and increase the weight;^8,9^ thus produces significant threats to aircraft safety. Whereas ice accumulation on a wind turbine can cause mechanical and electrical failures; results in up to 50% power losses.^10^ Several thermal, chemical and mechanical methods^8–11^ have been developed for eliminating the surface ice such as chemical fluid systems,^9,11^ pneumatic boots,^12^ electro-impulsive,^13^ hot air system,^11^ ultrasound system,^7,14^ microwave technology^15^ and electro-thermal de-icing system.^2^ Among these, electro-thermal de-icing technique is considered to be the most effective and energy efficient due to its ability to control the temperature and heat dissipation by Joule heating.^16^

Currently, fibre reinforced polymer composites are increasingly popular in aerospace, automobile and civil engineering industries due to their higher strength and lower weight.^17^ However, ice accumulation reduces the advantages that the composite brings to the structure. The electro-thermal system is identified as one of the most promising de-icing systems for polymer composites, as it does not cause delamination and damage to composite structure.^9,11^ However, the application of the electro-thermal system within composites is limited by the poor thermal conductivity and high thermal sensitivity of polymeric materials.^18,19^ Many studies^20–22^ have reported uses of conductive polymers, metals, CNT and carbon black to make conductive polymer composites; however they still suffer from poor thermal and electrical conductivity, and higher energy consumption. Therefore, it is desirable to use a conductive material that can provide excellent electro-thermal properties as well as can achieve desired temperature without compromising existing mechanical and thermal properties of composites.

Graphene, a single atom thick two-dimensional closely packed honeycomb lattice of sp^2^ carbon allotropes, has been focus of mass investigations in recent years due to its large surface area,^23^ record thermal conductivity,^24^ excellent mechanical strength,^25^ and superior electronic mobility.^26^ It has demonstrated much higher thermal conductivity ∼5000 W mK^−1^ than CNT (∼3000 W mK^−1^) and amorphous carbon (∼1 W mK^−1^).^27^ Previous study has demonstrated the use of graphene-based inks to produce highly conductive electrical conductor with lower resistance.^28^ Moreover, a small amount of graphene would be sufficient to form a percolative electric network due to higher aspect ratio and surface area;^29^ thus reduce overall weight of the composite. Recent studies have highlighted using of Graphene Nanoribbon (GNR),^2,30^ perfluorododecylated GNR^31^ and Graphene Nanoplatelets (GNP)^32^ for de-icing applications. However, there are number of problems still exist with these techniques such as complicated and expensive manufacturing route for GNR, not suitable for industrial applications and material waste. Moreover, strong van der Waals forces between graphene fillers may cause re-stacking in the polymer matrix, which may result in defective composites.^33^ Moreover, increased GNR concentration modifies the resin viscosity and increases the brittleness of composites.^34^

Here we report a scalable manufacturing route for next generation graphene-based de-icing composites. We utilise microfluidic exfoliation technique to synthesize graphene-based ink in a scalable quantity and use a dip-dry-cure coating technique to make highly conductive graphene-based glass fibre rovings. These graphene-coated glass rovings are characterised both electrically and thermally; then integrated into a composite structure. We demonstrate the use of as prepared composite for de-icing applications using Joule heating phenomena.

## 2. Experimental

### 2.1 Materials

The natural flake graphite (average lateral size ∼ 50 μm) was kindly supplied by Graphexel Limited, UK. Sodium deoxycholate (SDC) powder was purchased from Sigma Aldrich, UK. The glass fibre roving (Glass S, 758-AB-675) was purchased from AGY (USA). 290 gsm (grams per square meter) plain woven glass fabrics, EL2 Epoxy Laminating Resin and AT30 Epoxy Hardener were purchased from Easy Composites, UK.

### 2.2 Graphene exfoliation

We use microfluidization technique to exfoliate graphene in a scalable quantity following previously reported methods.^28,35^ Briefly, 50 g graphite powder and 10 g SDC are added into a glass bottle and mixed with 500 ml deionised (DI) water. This mixture is sonicated for 30 minutes using an ultrasound bath to allow homogenous dispersion and added into an input reservoir of a Microfluidizer (M-110P Microfluidizer, Microfluidics Corp, USA). The dispersion is slowly passed through ‘Z-type’ microfluidic channels of ∼200 μm and ∼87 μm diameter with diamond construction at high pressure (∼200 MPa). This allows the exfoliation of graphite to few-layer graphene (FLG) at 100 ml min^−1^ flow under high shear rate [∼10^8^ s^−1^] with a 100% exfoliation yield. The exfoliated dispersion is then passed through a cooling channel surrounded by cold water (∼25 °C) to prevent over-heating of the dispersion and collected. This process is repeated 20 times to produce FLG (MF flakes) which is then used as a conductive ink for glass roving coating.

### 2.3 Glass roving coating

We use a simple dip-dry-cure coating technique to coat glass fibre roving with graphene-based ink. The graphene-based ink is added into a 100 ml cylinder flask. Glass rovings are cut to 25 cm and dipped into graphene dispersion for 5 seconds. The coated glass fibre rovings are then passed through eyelets to squeeze extra ink out of coated glass tows and to maintain coating evenness. We then use a Mathis Laboratory dryer (Mathis, Switzerland) to dry (at 100 °C) and cure coated glass fibres. We also use various curing temperatures (110 °C to 240 °C at 10 °C interval), curing times (5 min to 30 min) and a number of coating cycles to optimise coating conditions.

### 2.4 Composite manufacturing

We use vacuum resin infusion process and room temperature thermoset EL2 epoxy resin to manufacture de-icing composites that contains graphene-based (coated) glass rovings. Briefly, 6 layers of glass fabric (dimension: 10 × 10 cm) are laid on a pre-cleaned and pre-coated (with a release agent) metal plate. The graphene-based glass rovings are then inserted after 3 layers of glass fabric and connected to wires for electrical and thermal characterisation. The sample is sealed by a plastic bag and vacuumed pressed using a pump. EL2 Epoxy Laminating Resin and AT30 Epoxy Hardener are degassed separately for 30 minutes and mixed together immediately before we use. The resin with hardener is then flown over layered glass fabrics at a constant flow rate using a vacuum pump, which enables the impregnation of glass fabrics with resin. The resin infused preform is then cured at room temperature for 24 hours to make graphene-based glass composites for de-icing application.

### 2.5 Characterisation of MF flakes and coated glass rovings

The graphene-based dispersion containing MF flakes is diluted 1000 times and then drop-casted on Si/SiO_2_ (290 nm oxide on plain silicon). The images and Raman spectra of MF flakes are taken at 10 different locations on the drop-casted sample to assess the flake size, flake thickness and flake types. We use an optical microscope to measure the flake size and Philip XL30 Field Emission Gun (FEG) Scanning Electron Microscope (SEM) to assess the surface topography of the untreated and coated glass rovings. A Dimension Icon (Bruker) Atomic Force Microscopy (AFM) is used to determine the flake thickness. A Renishaw Raman System equipped with 633 nm laser is used to collect Raman spectra of MF flakes, untreated and coated glass rovings. The surface of MF flakes, untreated and coated glass rovings is characterised using a Kratos Axis X-ray Photoelectron Spectroscopy (XPS) system. The thermal decomposition of graphite, SDC and MF flakes are evaluated using a TA instrument, TGA Q5000. The resistances of graphene-coated glass rovings per unit length are measured using a two probe multi-meter (DL9309 Auto Ranging Multimeter, Di-Log, UK). The average resistance is calculated from 5 measurements at different positions along the length of graphene-based (coated) glass fibre rovings.

### 2.6 Thermal analysis

The electro-thermal behaviour of coated glass fibre rovings is measured using a thermal camera (TIM 160, Micro-Epsilon, Germany) and a power supply unit (Stabilised Power Supply L 30D, Farnell Instruments LTD, UK) which supplies direct current at different voltages from 0–30 V. The graphene-based glass fibre rovings are clamped on a wooden plate using two metals clamps at 1, 5, 15 and 20 cm distances. A multi-meter is also used to measure the current. The thermal camera is installed on a tripod and connected to the computer. The thermal images obtained by the camera is shown on the computer through a software (TIM Connect, Rel.2.12.2202.0). The change in temperature of the coated glass is observed and recorded at various volts and currents. Thermal images of coated glass at various volts and currents are also captured.

## 3. Results and discussion

### 3.1 Graphene ink characterisation

We use microfluidization technique to exfoliate few layers graphene (MF flakes) from graphite into a water-based dispersion. Microfluidizer can pass fluids through micro-channels (diameter, *d* < 100 μm) at high pressure (up to 209 MPa),^35^ which generates liquid velocities of 400 m s^−1^ and several order of magnitudes higher shear rates (>10^8^ s^−1^)^36^ than conventional rotor-based or other homogenisers. It is used primarily for particle size reduction,^37^ nano-emulsion of immiscible liquids,^38^ for disrupting or lysing cells^39–41^ and de-agglomeration and dispersion of carbon nanotubes and graphene nano-platelets (GNP) into polymers.^41,42^ Recent studies have highlighted using microfluidization technique to produce graphene,^28,35^ graphene quantum dots^36^ and two-dimensional (2D) boron nitride nano-sheets.^43^ It is a simple and environmental friendly technique that can produce graphene-based ink with a 100% exfoliation yield.^28^ Moreover, this process could be scaled up to produce hundreds of kilos graphene-based inks, which would suit perfectly for industrial applications such as manufacturing of graphene-based multi-functional composites.

We use Graphexel 2736 grade graphite (average flake size < 50 μm) supplied by Graphexel, UK. Fig. 2a shows the average lateral size of exfoliated flakes after 20 cycles (MF flakes) is ∼1.45 μm. Flake thickness analysis by AFM shows ∼20% flakes are <10 nm (ESI, Fig. S1†). Fig. 2b shows Raman spectra of exfoliated MF flakes after 20 cycles, which is a typical for liquid-phase exfoliated graphene, with characteristics D peak at ∼1350 cm^−1^, G peak at ∼1582 cm^−1^ and an asymmetric 2D-band at ∼2730 cm^−1^.^28,35^ For XPS analysis, the exfoliated MF flakes without any surfactant is drop-casted onto a PEL paper and attached onto a carbon tape. Similarly, starting flake graphite is attached onto a carbon tape directly. The wide scan XPS spectra reveal only C1s and O1s peaks for both starting graphite and MF flakes, Fig. 2c. C/O ratio of starting graphite materials and MF flakes (without surfactant) are 24.91 and 24.84, respectively. High resolution C1s spectra also reveals similar peaks for both starting graphite and MF flakes, which is dominated by C–C/C=C bond in aromatic rings (∼284.6 eV), (ESI, Fig. S2a and b†). During microfluidization, further oxidation or chemical functionalisation is not evident from C1s spectra, which in agreement with the previous study.^28^ TGA analysis shows slight decomposition (∼2 wt%) of starting graphite above 750 °C; whereas MF flakes start to decompose at lower temperature (∼5.5 wt% up to 750 °C), may be due to the lower thermal stability of smaller MF flakes.^44^ As expected, SDC suffers significant decomposition around 400 °C.

**Fig. 1:**
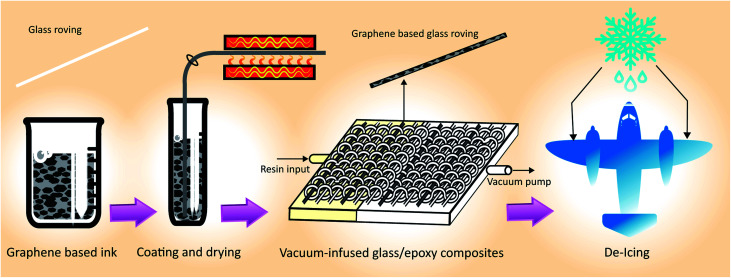
Process flow diagram for manufacturing graphene-based glass/epoxy composites for de-icing applications.

**Fig. 2:**
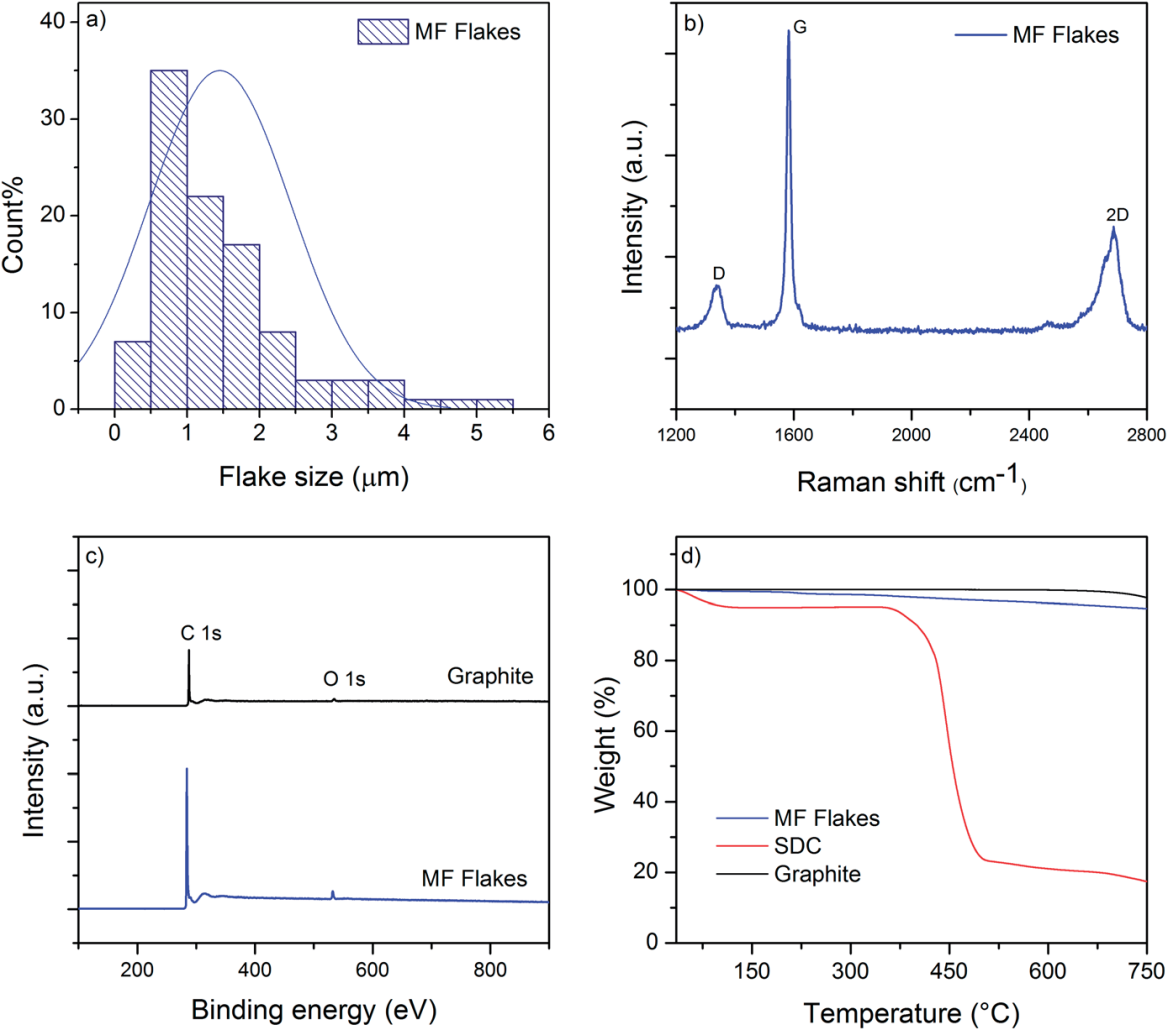
(a) The size distribution of MF flakes; (b) Raman spectra of MF flakes; (c) wide scan XPS spectra of starting graphite and MF flakes; (d) TGA curves of MF flakes, SDC and Graphite.

### 3.2 Glass roving coating, optimisation and characterisation

For electro-thermal de-icing application, it is desirable to produce highly conductive and uniform glass-fibre roving using a scalable process. We use a very simple dip-dry-cure technique that could potentially be scaled-up for industrial applications. As illustrated in Fig. 1, glass fibre roving is dipped into graphene-based ink for few seconds and passed through an eye-let of specific diameter to squeeze out additional materials from the surface; thus produce uniformly coated glass-fibre roving. The graphene-based glass roving is subsequently dried and cured at elevated temperature. These graphene-based (coated) glass-fibre rovings could be integrated into a composite by weaving, knitting or braiding and heated up to a desired temperature for de-icing composite applications.

**Fig. 3:**
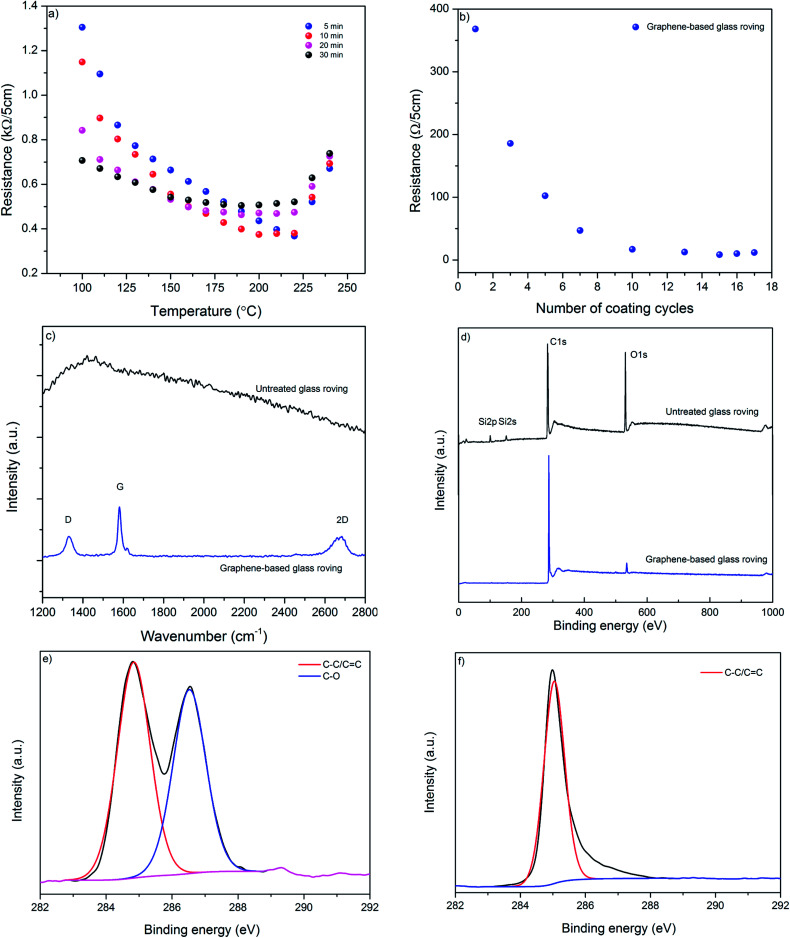
(a) The change of resistance of graphene-based ink coated glass rovings with time and temperature; (b) the change of resistance with number of coating cycles; (c) Raman spectra of uncoated and graphene-based glass rovings; (d) wide scan XPS spectra of untreated and graphene-based glass roving; (e) high resolution C (1s) XPS spectrum of control glass roving; and (f) high resolution C (1s) XPS spectrum of graphene-based glass roving.

Fig. 3a shows the effect of curing temperature and time on the resistance of coated glass-fibre roving. As expected the resistance per unit length of graphene-based glass-fibre roving decreases with the increase of temperature due to the thermal annealing of graphene flakes and the removal of residual solvents and surfactants.^45^ However, after a certain temperature, further increase in temperature results in slight increase in the electrical resistance of graphene-based glass fibre roving. This optimum temperature point for lowest resistance varies with the curing time, as higher curing time provides lowest resistance at lower optimum curing temp and *vice versa*. We use 220 °C for 5 minutes as optimum curing condition to enable rapid drying and curing in a continuous manufacturing process. Fig. 3b shows the change of resistance of graphene-based glass rovings with temperature. The electrical conductivity improves with the increase in number of coating cycles due to the deposition of higher amount of conductive MF flakes on the fibre surface and the formation of a continuous percolated network.^2,46^ Moreover, the contact resistance between graphene flakes are reduced with the increase of coating cycles. The lowest resistance of coated glass-fibre roving with graphene-based ink is obtained as ∼1.7 Ω cm^−1^ with 15 coating cycles.

Raman spectra of control glass roving shows smooth featureless background of uncoated fibre, which is masked by strong fluorescence, Fig. 3c. Whereas, graphene-based glass fibre roving shows characteristics graphene peaks at ∼1350 cm^−1^, ∼1582 cm^−1^ and ∼2700 cm^−1^, corresponding to D, G and 2D peaks, respectively. The D peak is caused by the defects and disorders in the hexagonal graphitic layers, while G peak attributed to an E_2g_ mode of graphite due to the vibration of sp^2^-bonded carbon atoms in a two dimensional hexagonal lattice.^47^ These Raman spectra are similar to the spectra of a typical liquid-phase exfoliated graphene or graphitic materials.^28,35,48^ The wide scan XPS spectra of control glass-fibre reveal the presence of C1s, O1s, Si2s and Si2p and provide the evidence of some silane coating on the fibre surface, Fig. 3d. However, after coating with graphene-based ink, C/O ratio significantly increased to ∼26.03 from ∼5.16 for control glass fibre.

The high resolution C1s spectrum of control glass-fibre can be fitted into two main components: C–C/C=C (284.6 eV) and C–O (286.4 eV), Fig. 3e. The coating with graphene-based ink enables diminishing of oxygen containing functional groups from the coated surface, Fig. 3f. For graphene-based glass rovings, C1s is mainly dominated by C–C/C=C, which is similar to graphene or graphite.^49^

**Fig. 4:**
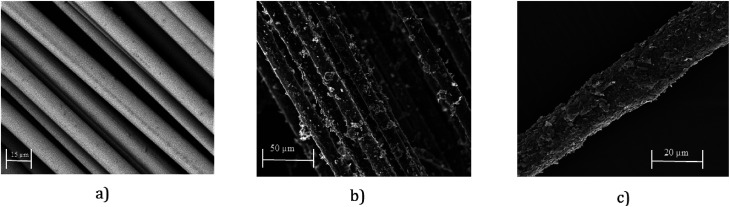
(a) SEM image of untreated glass fibre roving (×2000); (b) SEM image graphene-based (coated) glass roving (×500); and (c) SEM image graphene-based (coated) glass roving (×1000).

SEM images provide further evidence of the presence of graphene flakes, covering the glass fibre, Fig. 4b and c. SEM images of uncoated glass show smooth glass fibre, Fig. 4a. After coating with graphene-based ink, significant deposition of MF flakes on individual fibre surfaces is observed, Fig. 4b. The individual fibres are wrapped with MF flakes, Fig. 4c, and create continuous conductive track, which enables current to flow through the structure. The diameter of the fibres increases with the increase of number of coating cycles due to the deposition of more MF flakes on the surface.

### 3.3 Joule heating of graphene-coated roving

**Fig. 5:**
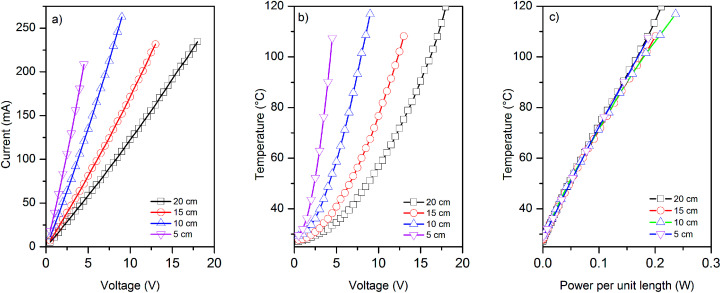
The change of (a) current and (b) temperature of graphene-based glass rovings at various voltages; (c) the power consumption per unit length of graphene-based glass rovings shows linear relationship with the increase of temperature.

We use four different lengths (5, 10, 15 and 20 cm) of graphene-based (coated) glass fibre rovings for electro-thermal characterisation and five samples for each length. We apply various voltages across the entire sample, and measure the current generated and the change in the temperature of graphene-based rovings (due to Joule heating) with voltages and the power consumed per unit length, Fig. 5a–c.

*I*–*V* curves for various length of graphene-based glass roving show the linear relationship between the applied voltages and current passes through conductive rovings, Fig. 5a. However, a non-linear relationship is observed for the change of temperature with voltages, Fig. 5b. The temperature of coated roving increases quadratically with voltage, as indeed expected. When the obtained temperature is plotted against the power per unit length (*p* = *IV*/*L*), the curves for the samples of different length collapse on the same straight line indicating that the major dissipation mechanism is the thermal radiation and convection rather than thermal conductivity through the contacts. Moreover, thermal images show uniform heat distribution along the length of conductive graphene-based glass rovings (ESI, Fig. S3†). Unlike Peltier effect, the heat is distributed over whole length of the conductor during Joule heating, which is also evident from the thermal images.

### 3.4 Joule heating of graphene-based glass-fibre composites for de-icing application

**Fig. 6:**
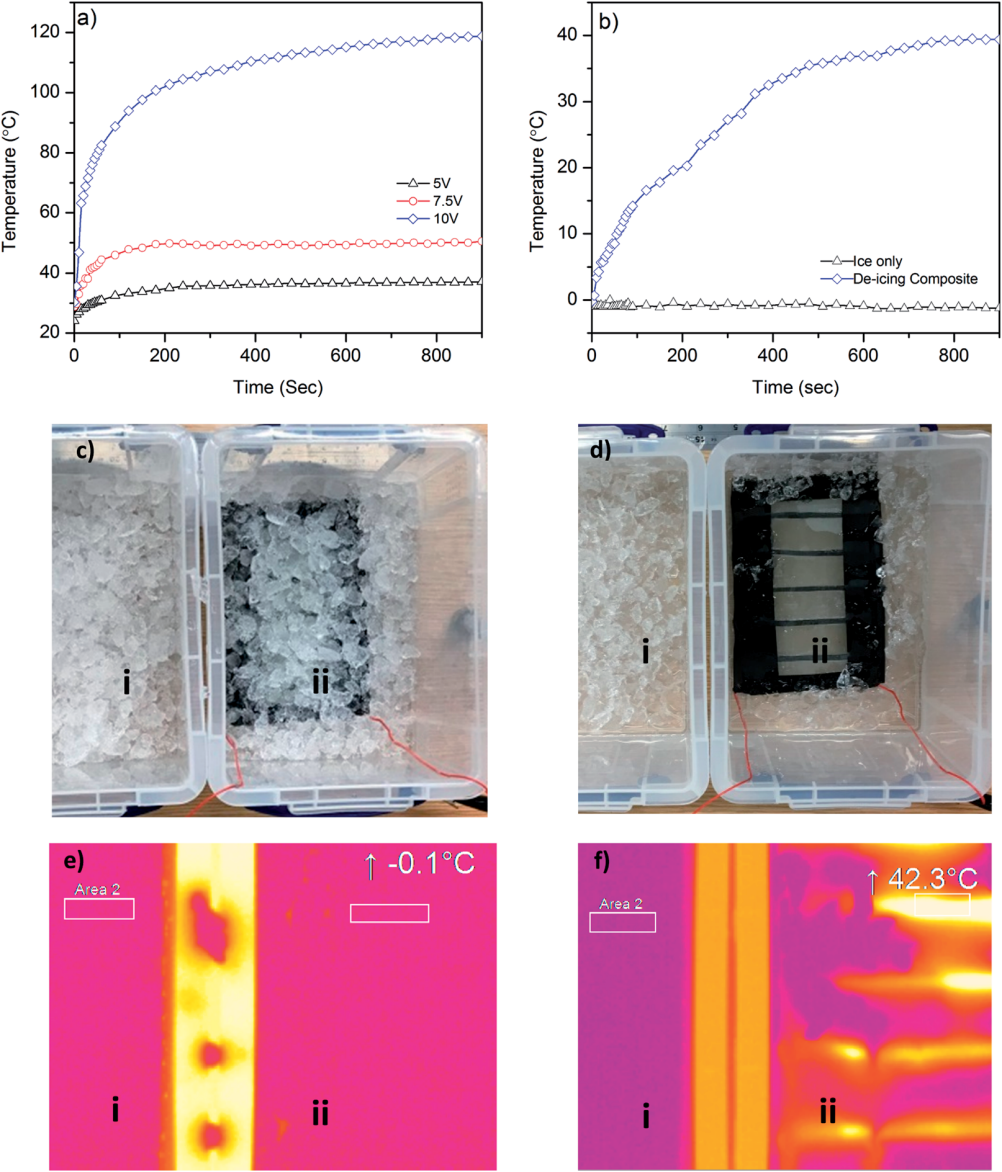
(a) Heating profile of graphene-based glass composite at various voltages (5 V, 7.5 V and 10 V); (b) the comparative change in temperature of only ice bucket and the ice bucket containing graphene-based de-icing composites. (c) Before heating: (i) ice bucket and (ii) graphene-based de-icing composite dipped into an ice bucket; (d) after heating at 10 V: (i) ice still in the bucket and (ii) removal of ice due to joule heating of de-icing composite; (e) before heating thermal images of (i) ice bucket and (ii) graphene-based de-icing composite dipped into ice showing similar temperature; (f) after heating thermal images: (i) ice still in the bucket and (ii) de-icing composite dipped into ice bucket demonstrates heating of the composites and removal of ice due to joule heating.

In order to demonstrate a potential de-icing application of graphene-based glass rovings, we manufacture a glass–epoxy composite using a vacuum resin infusion process, where five graphene-based glass rovings are inserted in the middle of 6 glass fabrics layers (ESI, Fig. S4a and b†). After making the composite, the graphene-based glass rovings are connected with external electrical wires and a power supply unit. At first, we investigate the time dependent temperature profile of the composite by applying voltages (5 V, 7.5 V and 10 V) across graphene coated glass rovings, Fig. 6a. The surface temperature of the composite increases with the increase of voltages, due to increase in the power. As shown in Fig. 6a, the temperature increases from room temperature (∼24.1 °C) to ∼36 °C after applying 5 V. Whereas for 7.5 V, the temperature increases rapidly from 30.3 °C to 51.8 °C within 30 seconds and becomes almost flat at 60 °C after 90 seconds. The temperature increases at much faster rate for 10 V, up to 71.6 °C within 30 seconds and continues to increase rapidly up to 100.8 °C after 180 seconds. After that slight increase in temperature is observed for 10 V. Similar time dependent temperature profile is observed in a previous study based on graphene nanoribbon (GNR);^2^ however at much higher voltages (20–40 V). Moreover synthesis of GNR is based on complex, time consuming and expensive process of unzipping CNTs.^50^ Furthermore, GNR was mixed with epoxy resin which may alter the rheology and uniformity of epoxy/hardener mixture and limit the concentration of conductive materials.^51^ In contrast, we use a scalable process for manufacturing graphene-based glass roving. Moreover, the coating of individual glass roving with graphene-based ink would enable insertion of coated warp or weft glass roving into the fabric or composite structure as required. This would potentially reduce the material waste and provide much better control and flexibility to remove various amount of ice from different parts of the structure.

Finally, we demonstrate the de-icing capability of our graphene-based glass/epoxy composites by dipping it into an ice-bucket, Fig. 6c. We then place this next to another bucket which contains only ice. We apply 10 V in order to see the de-icing effect of graphene-based composite. Fig. 6b shows rapid increase in the temperature and the melting of ice in the bucket containing graphene-based composites by Joule heating. The temperature increases from −0.1 °C to 27.3 °C within 5 minutes, whereas the temperature of the bucket containing only ice remains almost same (∼−1 °C) even after 30 minutes. Fig. 6d(ii) shows efficient removal of ice from the surface of graphene-based composite. Thermal image of both ice buckets shows similar temperature before applying heat, Fig. 6e(i and ii). The thermal image of ice only bucket shows almost similar temperature (<−1 °C) for 30 minutes. However, the temperature increases to 42.3 °C for the ice bucket containing graphene-based composite over that duration. This demonstrates the good perspective of our graphene-based composite for next generation de-icing applications.

## 4. Conclusions

We report a scalable process of manufacturing graphene-based surface heating for de-icing applications. The graphene-based glass roving demonstrates low resistance, ∼1.7 Ω cm^−1^ and efficient heating to a desired temperature at lower power consumption. We also demonstrate the use of this graphene-based glass rovings for manufacturing glass/epoxy composite for de-icing applications. The de-icing test shows efficient removal ice from graphene-based composite. We believe, the obtained results from this study would be an important step towards realising graphene-based next generation lighter, stronger and cost-effective smart de-icing composite for aerospace and other applications.

## Conflicts of interest

There are no conflicts to declare.

## Supplementary Material

RA-008-C8RA02567C-s001
