# Supplementary material for: Graphene-based surface heater for de-icing applications
Source: RSC Adv. 2018 May 8;8(30):16815–23. doi: 10.1039/c8ra02567c (PMC9080290; doi:10.1039/c8ra02567c)
Supplement: RA-008-C8RA02567C-s001 [file RA-008-C8RA02567C-s001.pdf]

Electronic Supplementary Material (ESI) for RSC Advances.
This journal is © The Royal Society of Chemistry 2018

Graphene-Based Surface Heating for De-icing Applications

Nazmul Karim,[1*] Minglonghai Zhang,[2] Shaila Afroj,[1,3] Vivek Koncherry,[2,4] Prasad Potluri,[2,4] and Kostya S Novoselov[1,3]

[1]The National Graphene Institute (NGI), The University of Manchester, Booth Street East, Manchester, M13 9PL, UK

[2]School of Materials, The University of Manchester, Oxford Road, Manchester, M13 9PL, UK

[3]School of Physics & Astronomy, The University of Manchester, Oxford Road, Manchester, M13 9PL, UK

[4]The Northwest Composites Centre, The University of Manchester, Oxford Road, Manchester, M13 9PL, UK

***Corresponding author E-mail:** mdnazmul.karim@manchester.ac.uk;

Keywords: graphene ink, de-icing composites, glass fibre, joule heating and smart composites

Supporting Information:

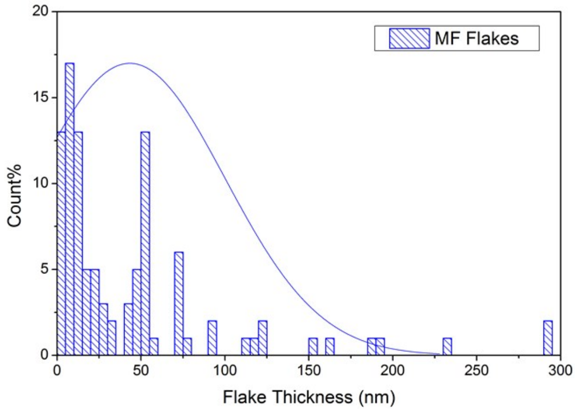

Figure S1. Flake thickness distribution of MF flakes

Supporting Information: High Resolution XPS spectra of Graphite and MF Flakes

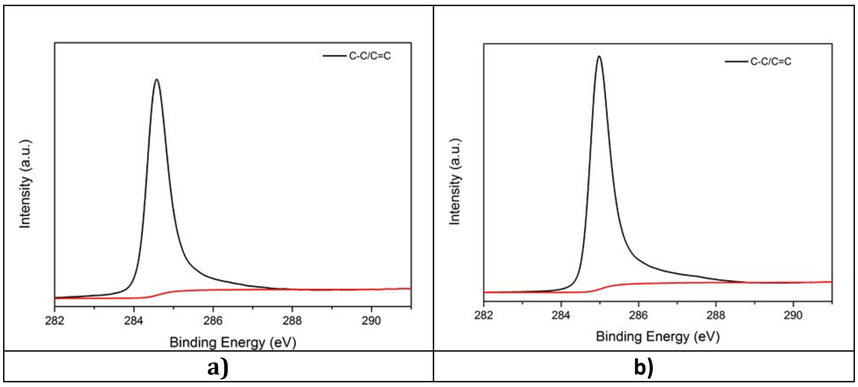

Figure S2. High resolution C (1s) XPS spectrum of a) starting graphite and b) MF flakes

Supporting Information: Joule Heating of Graphene-Based Glass Roving

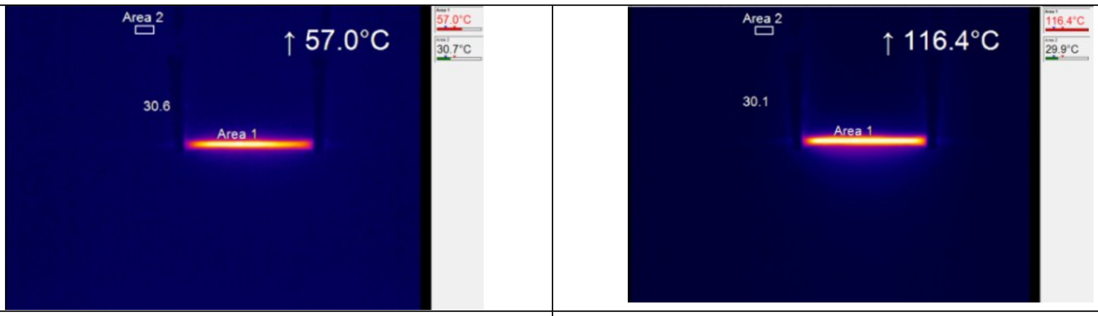

a)	b)

Figure S3. Thermal images of graphene-based glass rovings a) 3 V and b) 6V

Supporting Information: Graphene-Based Glass-fibre Composite

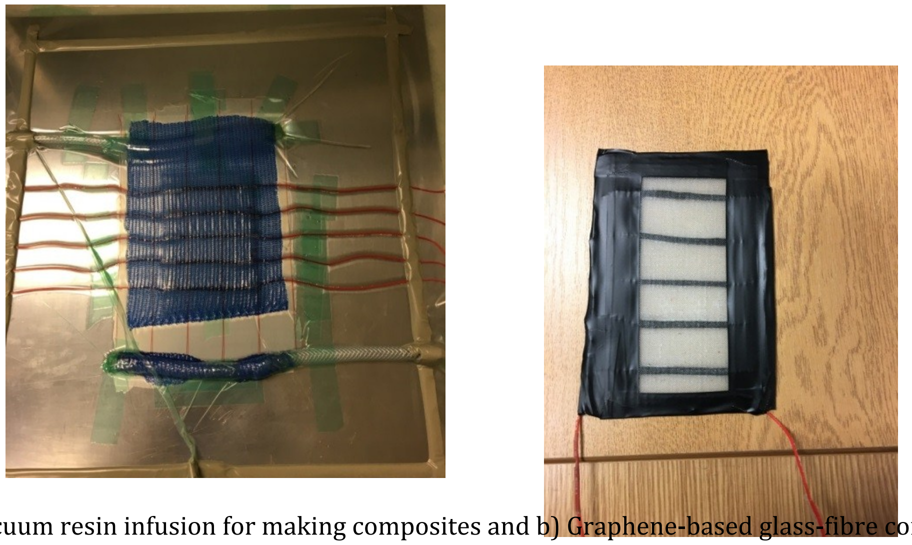

Figure S4. a) Vacuum resin infusion for making composites and b) Graphene-based glass-fibre composite for de-icing application
